# Evolution of Electroencephalogram Signal Analysis Techniques during Anesthesia

**DOI:** 10.3390/s130506605

**Published:** 2013-05-17

**Authors:** Mahmoud I. Al-Kadi, Mamun Bin Ibne Reaz, Mohd Alauddin Mohd Ali

**Affiliations:** 1 Department of Electrical, Electronic & Systems Engineering, Faculty of Engineering and Built Environment, Universiti Kebangsaan Malaysia, UKM Bangi Selangor 43600, Malaysia; E-Mails: mamun.reaz@gmail.com (M.B.I.R.); mama@eng.ukm.my (M.A.M.A.); 2 Department of Biomedical Engineering, Al-Khwarizmi College of Engineering, Baghdad University, Baghdad 47146, Iraq

**Keywords:** electroencephalogram (EEG), anesthesia, detection, signal processing, features, classification

## Abstract

Biosignal analysis is one of the most important topics that researchers have tried to develop during the last century to understand numerous human diseases. Electroencephalograms (EEGs) are one of the techniques which provides an electrical representation of biosignals that reflect changes in the activity of the human brain. Monitoring the levels of anesthesia is a very important subject, which has been proposed to avoid both patient awareness caused by inadequate dosage of anesthetic drugs and excessive use of anesthesia during surgery. This article reviews the bases of these techniques and their development within the last decades and provides a synopsis of the relevant methodologies and algorithms that are used to analyze EEG signals. In addition, it aims to present some of the physiological background of the EEG signal, developments in EEG signal processing, and the effective methods used to remove various types of noise. This review will hopefully increase efforts to develop methods that use EEG signals for determining and classifying the depth of anesthesia with a high data rate to produce a flexible and reliable detection device.

## 1. Introduction

Electroencephalography (EEG) is the neurophysiologic measurement of the electrical activity of the brain. Normally, this signal is a function of time and is described in terms of amplitude, frequency, and phase. The neurons communicate through electrical impulses and generate a bio-electromagnetic field that propagates through the brain tissues, skull, and scalp. The detectors are placed on the scalp to monitor signals from different locations at a time; these signals describe the brain activity. Many other methods are used for data acquisition, such as functional magnetic resonance imaging (FMRI) and positron emission tomography (PET), but EEG is the most popular method for assessing brain activity because of its simplicity, high temporal resolution, and low cost [1–3]. EEG recording technology is limited by the detection and characterization of existing nonlinearities in the surface of the scalp, estimation of the phase, acquisition of exact information, truncation of the noise from the signal, and classification of this signal.

Anesthesia is an indispensable part of surgery. Anesthesiologists monitor the depth of anesthesia (DOA) of patients based on observations on the underlying changes in physiologic symptoms, such as blood pressure, heartbeat, breathing rates, eye movement, and their physical responses to stimulation from the surgical procedure [4]. The features of the EEG signal vary with the level of anesthesia. This variation is utilized to monitor the depth of anesthesia. DOA is the dynamic balance between loss of consciousness and intensity of surgical stimulation. Unconsciousness is characterized by the lack of movement, awareness, and recall of the surgical intervention and unresponsiveness to painful stimuli, whereas the intensity of surgical stimulation depends on the type and duration of surgery [5]. Inadequate general anesthesia caused by underdosage causes intraoperative awareness with recall whereas prolonged anesthesia increases the risk of postoperative complications because of overdosage. The most important factor that contributes to the inadequate general anesthesia is the current limited ability to determine the level of awareness [6–8].

This paper provides a detailed review of the literature concerning the features and classifications used to recognize the stages of anesthesia from 1990 to 2012. It also briefly explains EEG signals and provides a short historical background of signal analysis. It highlights recent detection, decomposition, and processing methods related to DOA. The review aims to discuss the stages for developing an ideal method for monitoring DOA and provides a good background regarding the challenges and problems in developing appropriate solutions to the outstanding issues.

## 2. History of EEG Signal Processing

Researchers have focused on brain signals since the beginning of the last century and several attempts to understand and interpret those signals have been proposed. Exploring brain signals underwent several stages that profoundly affected the interpretation of brain signals during anesthesia. The purpose of reviewing these general methods for detecting and classifying brain signals is to show the efforts that helped to find efficient methods for monitoring the patients during surgery.

### 2.1. Emergence and Development of General EEG Signals

In 1875 the English physician Richard Caton discovered the presence of electrical current in the brain [9–11]. He observed continuous and spontaneous electrical activity from the brain surfaces of rabbits and monkeys. In 1912, Russian physiologist Vladimir Vladimirovich Pravdich-Neminsky published the detection of the first brain signals and evoked potentials in mammals (dog). Fuller said that [12] the German neurologist Hans Berger recorded the first human brain signal in 1924. They used ordinary radio equipment to amplify the brain's electrical activity and recorded it graph paper. The scientist named the device “EEG”. Berger also noticed that rhythmic changes in the brain waves varied with the state of consciousness of the subject. Franklin Offner developed EEG equipment and introduced concentric needle electrodes [13]. In 1935, Gibbs *et al.* described the characteristic form of spike waves, which started the field of clinical electroencephalography [14]. Subsequently, in 1936, Gibbs and Jasper reported the interictal spikes as the focal signature of epilepsy [15,16]. After World War II, the researchers tend to develop different methods of detection, purification, and classification of brain signals that enabled them to diagnose abnormal signals. In the 1950s, English physician William Grey Walter developed EEG topography, that allowed for the mapping of electrical activity across the surface of the brain; this topography was used in psychiatry until the 1980s. From 1990 to 2000, many techniques were developed to process EEG signal such as Blind Source Separations (BSS) [17–22] and Independent Component Analysis ICA [23–25]. The neural network detection systems, proposed in 1996, are used to classify EEG signals according to the feature of the recorded signal; some of these features will be explained in detail in subsequent sections [26,27].

### 2.2. Emergence and Development of EEG Signals during Anesthesia

Measuring the depth of anesthesia uses most of the previous methods, which are being continuously improved. In 1847, John Snow described five levels of anesthesia, which Guedel later refined into four stages based on somatic muscle tone, ocular signs, and respiratory parameters [28]. In 1957, Woodbridge described four stages of anesthesia from another point of view, *i.e.*, sensory blockade, motor blockade, blockade of autonomic reflexes, and loss of consciousness [29]. In 1991, van de Velde and Cluitmans evaluated the characteristic frequencies in the EEG data from a cat. They tried to assess the anesthesia levels by calculating the EEG spectra. They found that the “spectral edge frequency” is a promising EEG parameter for assessing the anesthetic depth [30]. In 1994, Watt *et al.* examined EEG signals as a non-linear dynamic system and classified EEG signals into three stages: light, nominal, and deep anesthesia. These researchers found that sufficient doses of anesthetic decrease the dimensionality of EEG samples with increasing anesthetic depth. This property is useful for classifying the activity of the brain during anesthesia [31].

Gugino *et al.* identified the changes in anesthesia induction using a combination of sevoflurane, propofol, and remifentanil. The results showed that light sedation accompanied by decreasing posterior alpha waves and increasing the intensity of frontal/central beta waves [32]. The fuzzy classifier is trained to define the anesthesia states: awake, moderate, general anesthesia, and isoelectric. The classification results were better than those of other methods using single features and systems that completely discriminate between awareness and general anesthesia state [33].

The researchers reduced EEG dimensionality by utilizing algorithms. One of these methods is called Isomap, which is based on estimating the phase of the continuum using the data features calculated from EEG sequences during deep anesthesia. Using the results from a one-dimensional feature, this method assesses neurophysiologic changes during anesthesia and provides a potential for developing more advanced systems for determining the depth of anesthesia [34]. Finally, to minimize the time required for interpreting EEG signals, many researchers suggested a common approach for extracting a single invert value that represents the patient's depth of anesthesia using the normalized bispectral ratio. This approach is based on the difference between the bispectral values of EEG signals during conscious and unconscious states in humans. The results showed a high capacity for distinguishing levels of consciousness with a simple numerical value rather than graphical presentations of levels of consciousness [10,35–37].

## 3. Background of EEG Signals

To analyze the brain signals during anesthesia we need to understand the properties of EEG signals such as frequencies, amplitudes, and internal and external effects that change the shape of these signals.

### 3.1. Mathematical Representation of EEG Signal

Many devices are used to process various kinds of biosignals, such as EEG, electromyogram (EMG), electroneurogram (ENG), electroretinogram (ERG), electrooculography (EOG), and electrocardiogram (ECG) to diagnose diseases [38]. These devices use the nervous system, which consists of a large number of excitable connected cells called neurons that rapidly and specifically communicate with different parts of the body through electrical signals. The nervous system consists of three main parts: the brain, the spinal cord, and peripheral nerves. It functions to controls the body and communicates through electric signals [39]. The brain signals are acquired using electrodes mounted directly on the scalp. The combination of these signals is illustrated in Equation (1) [21]:

(1) $$X(t)={\left[X_{1}(t),X_{2}(t),\ldots ,X_{m}(t)\right]}^{T}$$

where *X*(*t*) is the recorded EEG signal, “*T*” denotes transposition and “*m*” is the number of channels. The rows of the input matrix are EEG signals recorded at different electrodes, whereas the columns represent the variations in the signals at different time points. Before the EEG signal is displayed or stored, it can be processed to eliminate low-frequency or high-frequency noise and other possible artifacts. The user is frequently interested in the amplitude of the signal; hence, critical points in its processing need careful treatment to reduce artifacts that contaminate signals, which can lead to wrong results and conclusions. Equation (2) shows a model that represents the recorded mixed EEG signal *X*(*t*) with time, varying source signal *s*(*t*), and mixing matrix *A* added to the external noise *n*(*t*). Considering only the *X*(*t*) is available, several assumptions are needed to estimate the matrix “*A*” and the signal *s*(*t*) [40,41]:

(2) $$X(t)=As_{m}(t)+n_{m}(t)$$

### 3.2. Characteristics of EEG Wave Bands

The EEG signal is traditionally divided into spectral broad frequency bands related to EEG generators and rhythms: delta, theta, alpha, and beta.

1. Delta (**δ**): This wave is generated from the thalamus, with a frequency signal range of up to 4 Hz and amplitudes ranging from 20 μV to 200 μV. This wave is often associated with young patients, certain encephalopathies, and underlying lesions. It is seen in the deep stage of sleep.
2. Theta (**θ**): This band is generated from the hippocampus and neocortex, with frequencies ranging from 4 Hz to 7 Hz and amplitudes ranging from 20 μV to 100 μV. This band is associated with drowsiness, childhood, adolescence, and young adulthood.
3. Alpha (**α**, Berger's wave): This band is generated by the thalamus, with frequencies ranging from 8 Hz to 12 Hz and amplitudes ranging from 20 μV to 60 μV. It is a characteristic of a relaxed, alert state of consciousness. Alpha rhythms are detected with the eyes closed and it attenuates drowsiness and open eyes, which can be seen over the occipital (visual) cortex.
4. Beta (**β**): This band is generated from the cortex, with frequencies ranging from 13 Hz to 30 Hz. This signal has a characteristically low amplitude (2 μV to 20 μV). Multiple and varying frequencies are often associated with active, busy, or anxious thinking and active concentration. Rhythmic beta waves with a dominant set of frequencies are associated with various pathologies and drug effects.
5. Gamma (**γ**): have frequencies ranging from 30 Hz to 70 Hz and very low amplitudes (3 μV to 5 μV). Some researchers classify this band as beta waves because they have similar properties [29,42,43]. The variations in the EEG signal bands during anesthesia are discussed in detail in Section 4.4.

### 3.3. Noise and Factors Affecting to EEG Signal Bands

The dynamic ranges of the EEG signal are usually ±100 μV before amplification. These signals acquire many types of noise when they travel through different tissues. The characteristics of the noise affect the value and shape of the EEG signals. These are classified into the following types:

- Inherent noise: The electronic equipment generates noise that overlaps with the recorded EEG signal. This noise can be eliminated by high-quality electronic components of the EEG recorder.
- Ambient noise: Radiation from electromagnetic devices is the main source of this noise. The ambient noise has greater amplitudes than the EEG signal. A shielded room should eliminate this type of noise.
- Motion artifacts: When these artifacts overlap with the EEG signal, the information signal is skewed and irregular. Motion artifacts have many sources: (a) Electrode interface; (b) electrode cable; (c) ocular artifacts; (d) swallowing; (e) sweating; and (f) breathing. Motion artifacts can be reduced by properly designing the electronic circuitry and using a smart program that separates and removes these artifacts from the EEG signal.
- Inherent signal instability: The amplitude of the EEG signal is naturally random. ECG artifacts affect the EEG signal especially the amplitude of the ECG signal changes during the different stages of anesthesia. ECG artifacts occur because of the cardiac electrical field that affects to the surface potential near the scalp. Smart programs should suppress these artifacts from EEG signal [44,45].

On the other hand, many factors affect the recorded EEG signal. These factors are categorized as follows:

- Causative Factors: This factor directly affects the recorded EEG signals and are classified as follows:
  - Extrinsic: This factor is due to the electrode structure and placement such as the shape of the electrodes, detection surface, distance between electrode detection surfaces, and location of electrodes with respect to the scalp volume.
  - Intrinsic: Anatomical, physiologic, and biochemical factors caused by the number of active motor units, nerve type composition, blood flow, nerve diameter, depth and location of active nerve, and the amount of tissue between the surface of the scalp and the electrode.
- Intermediate Factors: These are physiologic and physical phenomena influenced by one or more causative factors. Interference from nearby nerve is an example of an intermediate factor.
- Deterministic Factors: These are influenced by intermediate factors. The number of active motor units and mechanical interaction between nerves directly affect the information in the EEG signal and recorded force [46].

Depth of anaesthesia is hard to assign, as increasing the concentration of anesthetic is associated with the various phenomena such as loss of cognitive ability and amnesia, these phenomena are balanced against the intense arousal that surgical stimulation can induce. The challenges and difficulties of EEG acquisition during anesthesia concentrate in the quality of the data. As a matter of fact, the recorded EEG data are influenced by external or internal sources of electromagnetic waves as we mentioned above. This is the main reason for the limited value of raw EEG records to monitor the depth of anesthesia.

## 4. Anesthetic Agents and Monitoring General Anesthesia

Before discussing the anesthetic agents and their effects on the patient, as well as the stages of general anesthesia, awareness should be defined. Awareness during general anesthesia is defined as the degree of awareness, reflected by the occurrence of evident or implicit memory of intraoperative events [5,29]. Based on this definition, the researchers in the field of pharmacy produced many kinds of anesthetics that are compatible with the type of surgery and the patient.

### 4.1. Anesthetic Agents

Anesthetics agents that induce general anesthesia are classified into intravenous agents and inhalational agents (volatile).

#### 4.1.1. Intravenous Anesthetic Agents

Intravenous Agents are administered with sedatives or narcotics. The depth of anesthesia peaks rapidly (causing loss of consciousness) and then decreases as the plasma concentration of the anesthetic declines because of the rapid redistribution of the drug. Table 1 illustrates the advantages and disadvantages of widely used intravenous anesthetic agents. The use of intravenous sedative-hypnotics became more common with the introduction of propofol. The clinical effects of a particular anesthetic concentration vary among patients. Specifying the type of anesthetic agent depends on the patient status, age, the time required to complete the surgery, and the type of surgery [47–50].

#### 4.1.2. Inhalational Anesthetic Agents

Inhalational agent (gas or volatile) causes the immobility through action at the spinal cord level and loss of consciousness at the supraspinal and cortical levels. Table 2 shows the most important volatile agents. Considering the amount of absorbed anesthetic depends on both time and body mass, the speed percentage of anesthetic agent (slow, fast, and very fast) depends on their solubility in the blood [51–55].

### 4.2. Monitoring the Depth of Anesthesia

Accurate assessing the depth of general anesthesia induced by intravenous agents is very difficult. The concept of minimum infusion rate (MIR) proposed by Sear *et al.* was used to compare the anesthetic requirements of intravenous agents during total intravenous anesthesia factor (TIVA). The researchers calculated the dose at which the agent was 50% effective (ED_50_) and 95% effective (ED_95_) and compared them with the analogous inhalational unit called minimum alveolar concentration (MAC). The MIR is greatly affected by the properties of the drug, and the age and physical status of the patient [37].

Many indices are used as references for monitoring the depth of anesthesia. Most of these indices are based on the changes in EEG signal with intravenous agents, whereas others depend on measuring the MAC with the inhalational agents. These indices are shown in Table 3. We will discuss the details of the three most commonly used devices; the other indexes are slightly different in terms of construction and algorithms [10,56–62].

#### 4.2.1. Bispectral Index (BIS)

The BIS index was first introduced in 1992 by Aspect Medical Systems. BIS is a statistical index based on a combination of time, frequency domain, and high-order spectral subparameters. Large volumes of clinical data are utilized to generate a single variable based on the disparity of EEG signal; this disparity correlates the behavior of sedation and hypnosis. BIS ranges from 100 (when the patient awake) to zero [10,63,64]. Generally, the bispectral index is computed in two steps:

1. Finding the discrete Fourier transform (DFT) coefficients.
2. Computing the bispectrum using the following equation [65]:

(3) $$B(f_{1},f_{2})=X(f_{1})\times X(f_{2})\times X*(f_{1}+f_{2})$$

where B(*f*_1_, *f*_2_) is the complex bispectrum and *X*(*f*) is the complex Fourier transform at frequency *f* of the EEG signal *x*(*n*) The bicoherence is utilized to find the relationship between the power at two frequencies *f_1_* and *f_2_* in one EEG signal; bicoherence can be computed separately for each electrode.

Loss of consciousness occurs at values between 70 and 80. The values that reflect adequate hypnotic effect are from 40 to 60, which correspond to the general anesthesia. BIS indices less than 30 represent deep anesthesia (patient at risk). Hence, the anesthesiologist must adjust accordingly to increase this value. BIS is useful for adjusting the dosage of anesthetics; this adjustment prevents any disturbances in the patient's situation (awareness or suppress EEG signal).

**Figure 1.**
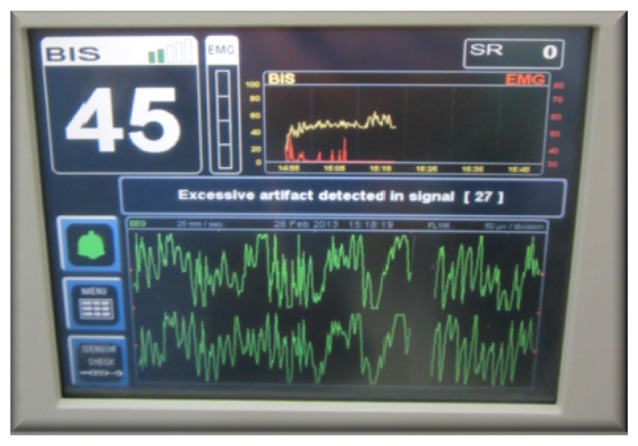
The BIS VISTA Monitoring System from Covidien.

BIS have limitations, BIS is an indicator for cortical activity that does not directly reflect the activity of the spinal cord and cortical structures; thus, this index may not reliably predict the responsiveness of harmful stimuli. Puri GD explained that the inaccuracy of BIS index is associated with the presence of senile dementia [35]. Other factors may confound the interpretation of BIS when the anesthesiologists use ketamine and N_2_O, wherein the BIS index may increase or may not change even when the patient is unconscious. This discrepancy is because BIS detects cerebral hypoperfusion, which is concomitant with anesthesia, particularly when combined with ketamine, propofol, and fentanyl [66–68]. Therefore, BIS monitoring devices do not reliably assess the DOA, especially when ketamine–nitrous oxide is used. Finally, the BIS algorithm is constantly updated by many companies such as “Covidien”, which supplies several BIS devices for monitoring the DOA based on two or four channels to acquire EEG signals (Figure 1).

#### 4.2.2. Narcotrend Index NCT

**Figure 2.**
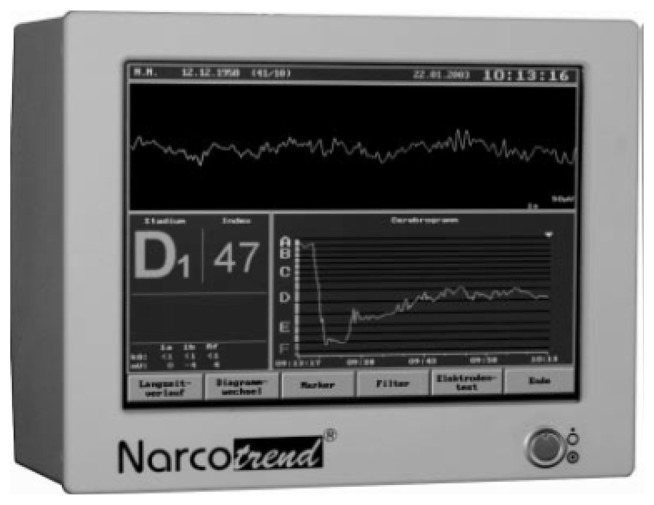
Frontal view of the Narcotrend Monitoring Device.

The Narcotrend is an EEG monitor produced by MonitorTechnik and developed at the University Medical School of Hannover, Germany. It is designed to measure the DOA and was introduced in the year 2000. Figure 2 shows the commercial Narcotrend monitor device. The Narcotrend algorithm is based on the work of Loomis *et al.* [69]. The raw EEG signal is recorded by a single or double-channel. After denoising and Fourier transformation (FT), the algorithm state six stages of anesthesia, as presented in Table 4. The system included a series of substages, resulting in 14 possible substages.

The alphabet scale in Table 4 has been translated into a numerical scaling index similar to BIS scale system (0 to 100), called the Narcotrend index. This is a scale that is quantitatively similar to BIS scale, ranging from 100 (awake) to 0 (deeply anesthetized).

Kreuer *et al.* [70] compared the NCT index with the BIS index and found sufficient correlation between these two indices, but are not identical in some ranges. Therefore, direct conversion from BIS to NCT values is not adequate. The Narcotrend index is a good DOA indicator in children when using sevoflurane and propofol/remifentanil [71,72]. Russell found that NCT is unable to differentiate conscious and unconscious patients during general anesthesia when neuromuscular blocking agents are used [73].

#### 4.2.3. Entropy

**Figure 3.**
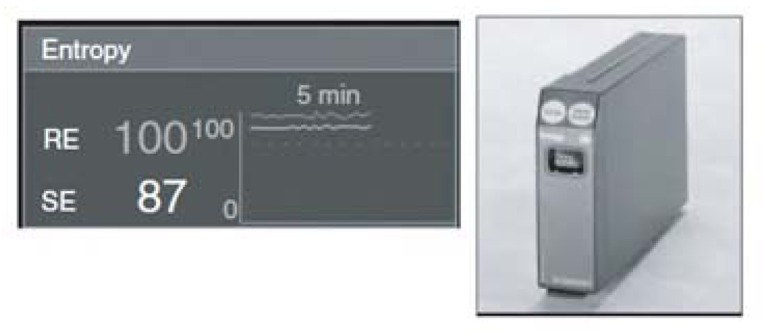
Entropy monitoring device and partial screen.

The Entropy system was introduced by the Datex-Ohmeda Company in 2003. The Entropy monitoring algorithm was designed to acquire and process raw EEG data and the frequency of EMG signals. Intensive studies by several researchers have led to the adoption of the system for monitoring DOA [74,75]. The numerical scale of entropy is similar to that of BIS and NCT, ranging from 0 (awareness) to100 (deep anesthesia). This system calculates the entropy in two numerical values: the first is the response entropy (RE), which has a maximum of 100 and includes information from EEG; the second is state entropy (SE), which has a maximum of 91 and includes the EMG activity. Figure 3 shows the Entropy module, with a partial screen that includes RE and SE.

The concept of entropy assumes that increasing DOA corresponds to increasing regularity of the EEG. The power spectra of certain epochs of EEG signals are used to calculate the spectral entropy. The second step is calculating the spectral entropy from the power spectrum of the EEG signal within a particular frequency band. A difference of 0 to 3 between RE and SE indicates adequate anesthesia. An increase in the difference between these two values provides a good indication for increasing the activity of frontal muscle (increased activity of EMG signal), which is a sign of inadequate anesthesia. As the anesthesia wears off, the effect of the drugs on the nervous system diminishes, which can be observed as activation of the frontal muscle. This monitoring has been validated for desflurane, sevoflurane, propofol, and thiopental. Entropy has not tested with ketamine [61,76,77].

#### 4.2.4. Minimum Alveolar Concentration (MAC)

In 1965, Eger *et al.* defined MAC as the minimum alveolar concentration of inhaled agents required to prevent 50% of subjects from responding to standard painful stimuli with gross purposeful movements [78]. After 10 years, this indicator has been expanded to the following:

(1) MAC-Intubation: prevent movement and coughing during intubation.
(2) MAC-Incision: inhibit movement during initial surgical incision.
(3) MAC-bar: inhibit adrenergic response to skin incision.
(4) MAC-awake or ED_50_: prevent response to verbal commands [79,80].

The MAC curves represent the relationship between the concentration of the agents and the probability of response. Hemodynamics responds to harmful stimulation and do not correlate well with decreasing drug concentrations. Consequently, the relationship between movement (somatic) and hemodynamic (autonomic) responses is poor during inhalational anesthesia. MAC provides the best method for monitoring the concentration of inhalation anesthetics to prevent movement (1.3 times the ED_50_) and to provide equilibrium among the alveoli, blood, and effect site. MAC increases because of alcoholism, hyperthyroidism, and hyperthermia, and decreases with increasing age, pregnancy, hypothermia, hypoxia and acidosis, severe hypotension, and sedative drugs including a2-agonists, ketamine, and intravenous local anesthetics [37].

#### 4.3. Evoked Potentials (EPs)

Another method for monitoring the DOA is EP. This method is based on stimulating specific areas and recording the responses in the brainstem, midbrain, and cerebral cortex. EPs represent the relationship between time and voltage, which is quantified by measuring the amplitude of the waveform during post-stimulus latency and interpeak. Three types of EPs were investigated for monitoring DOA [81].

- Somatosensory EP (SEP): records the response to stimulation over the somatosensory cortex (peroneal, tibial, or median nerve).
- Visual EP (VEP): records the response to photic stimulation (using flashing lights to the eyes) over the occipital cortex. This technique has been used to monitor functions during surgery for lesions involving the optic nerve, pituitary gland, and the optic chiasma.
- Auditory EP (AEP): records the response to auditory cortex stimulation (audible clicks) to the auditory canal.

Jeleazcov *et al.* [82] combined two kinds of simultaneous monitoring methods—AEP and SEP—and compared the result with EEG signals. He defined four levels of general anesthesia: awake, light anesthesia, surgical anesthesia and deep surgical anesthesia. The results showed that the discriminant power of EEG variables is more significant than AEP and SEP variables to define the four levels of anesthesia. In same context, the researcher found that EEG and AEP give a higher representation for general anesthesia than the information acquired from EEG alone. AEP is most commonly used for assessing DOA and is divided into three main parts (brainstem, middle latency, and long latency), which depends on the time and the site of origin [83,84]. Most of the inhalational and intravenous agents increase brain stem latency, which is directly proportional to increasing DOA. Using EP to monitor DOA requires additional techniques to record the EPs. Many types of artifacts can distort the EP, such as stimulus characteristics (duration and intensity), anesthetic drugs, electrode placement, age, and gender [85].

Recently, there have been vigorous attempts by researchers to find a new index to be able to determine the level of sedation with the drug concentration change. Li *et al.* constructed a new index (SI) using the entropy of the eigenvalues of the cortical coherence for each pair of channels as a feature to find the effect of sevoflurane, desflurane, isoflurane, and enflurane during general anesthesia in sheep. They found a significant correlation between the increase in spatial and anesthetic-induced cortical depression as well as SI succeed to measure cortical synchrony during general anesthesia [86]. Liang *et al.* explored the dynamical features of brain activity during anesthesia using permutation auto-mutual information PAMI method. Information coupling in EEG series can be applied to indicate the effect of the anesthetic drug sevoflurane on the brain activity, as well as other indices. This method was proposed to measure the information coupling of EEG time series under sevoflurane anesthesia. The PAMI of the EEG signals is suggested as a new index to track drug concentration change. This model is assessed by pharmacokinetic/pharmacodynamic (PK/PD) modeling and prediction probability. Pharmacokinetic are the actions of drugs within the body, as their distribution, absorption, elimination, and metabolism where pharmacodynamic are the relating to drug action at the receptor level. The researcher found that the PAMI index correlates closely with the sevoflurane anesthetic agent [87].

### 4.4. The patient Under Anesthesia

General anesthesia consists of four components, namely:

(1) Amnesia (lack of memory).
(2) Analgesia (lack of pain).
(3) Hypnosis (lack of response).
(4) Muscle relaxation.

All these components occur at once depending on the concentration of the agent. Then, the patient goes through different stages of anesthesia. Guedel was the first to identify the four stages of general anesthesia [88]:

Stage 1: Analgesia and amnesia:
  (A) The patient is wheeled to the recovery room.
  (B) Memory is slow to return.
  (C) Breathing is regular but slow.
  (D) Patients can converse, but have no memory of what you say.
Stage 2: Delirium and unconsciousness:
  (A) The patients are at highest risk for laryngospasm.
  (B) Patients breathe unassisted, but are not able to defend their airways.
  (C) Breathing is irregular. At this stage, the patient seems to be breathing and ready for extubation, but we must wait until patients are able to respond to commands: The patient has to prove they are in stage 1 before they can be extubated.
Stage 3: Surgical anesthesia:
  (A) The goal before starting surgery.
  (B) Patients breathe on their own if no muscle relaxants are given.
Stage 4: Overdose (stops breathing):
  (A) If more anesthetic is given.
  (B) Blood pressure continues to fall until circulatory collapse occurs. This result is due to inhibition of the cardiorespiratory centers in the medulla.

All patients undergo these four stages; however, some patients require more anesthetic than others to achieve a given response.

### 4.5. Characteristics of EEG Signal during General Anesthesia

Increasing the drug concentration directly affects the amplitude and frequency of EEG signals. This variation depends on the type of anesthetic and the age of patients. At the beginning, lower doses of anesthetic essentially increase the amplitude of the beta band in the frontal regions (frequencies exceeding 20 Hz) and decrease the amplitude of the alpha band. The eye movement artifact appears clearly during this stage. When the anesthetic concentration is increased to the surgical level, the frequency of theta and delta bands decrease, whereas their amplitudes increase. Further increases in the anesthetic concentration generate a special EEG pattern known as burst suppression (BS). Alternating periods of high amplitude and low voltage is the main feature of this pattern. Any further increases in the anesthetic dose cause suppression and electrical silence. Finally, the induction of anesthesia associated with the frontal portion of the brain with increased beta activity and delta activity appeared in the posterior regions and migrates toward the frontal regions [3,32,89].

## 5. EEG Signal Processing

**Figure 4.**
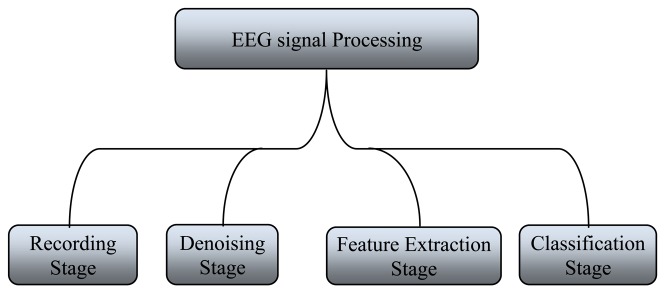
The main stages that use to process EEG signal.

General signal processing methods are used to process EEG signals during anesthesia with some modification. EEG signal analysis undergoes four stages as follows: recording stage, dancing stage, feature extraction stage, and classification stage. These processes are summarized in Figure 4, where each stage is discussed in detail. The implementation of these stages must be sequential, starting from the recording stage to the classification stage. At each stage, several operations should be carried out before sending the signal to the next stage.

### 5.1. EEG Signal Recording and Detection

**Figure 5.**
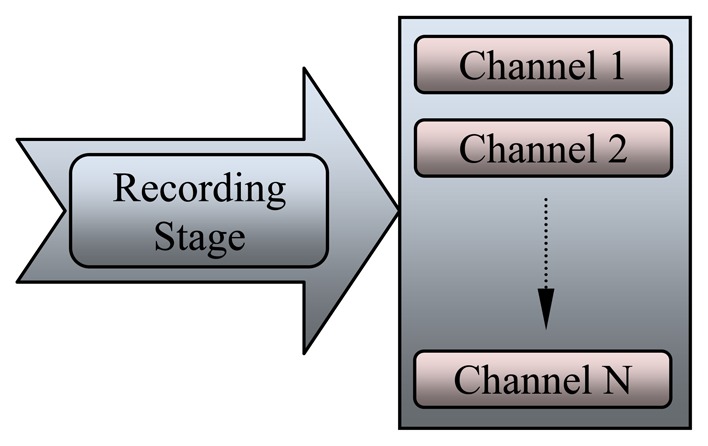
The multi-channel recording stage.

Precise recording and detection of discrete events in the EEG signal is an important issue in EEG data analysis. Figure 5 shows the recording stage, which consist of many channels (electrodes) that collect EEG at different locations.

**Figure 6.**
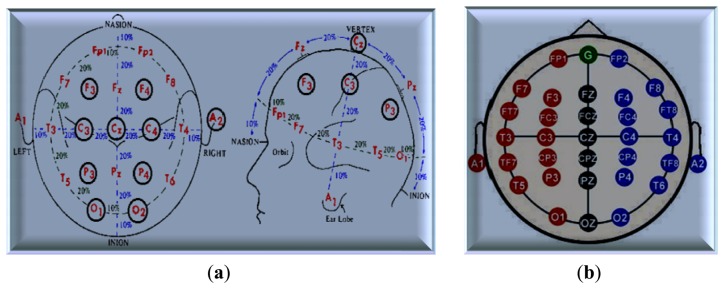
The general distribution of electrodes using International 10–20 EEG system and their name (**a**) The apportionment of the electrodes; (**b**) the distribution of the electrodes

Several methods have been proposed to record and collect EEG signals according to electrode type, number of electrodes (number of channels), position of these electrodes, and purpose of the recording signal. Before placing the electrodes, the skin should be prepared with alcohol and wiped with a special gel that helps increase the electrical conductivity of the electrodes (acceptable impedance below 5 kΩ) [90]. The number of channels depends on the number of electrodes affixed onto the scalp, varying from 1 to 20. The international 10–20 system depends on the size of the head, which is divided into several areas, as shown in Figure 6(a). Some researchers use two to four electrodes in the frontal region to record the EEG signals to detect the DOA. Zoubek *et al.* used the 10–20 EEG system with four channels to record the EEG signals at the following locations: C3–A2, P3–A2, C4–A1, and P4–A1, as shown in Figure 6(b), with additional transversal EOG, one chin EMG signal, and a 128 Hz sampling frequency [91].

Other groups used the international 10–20 system to record EEG signals and analyze the bispectral index scale (BIS) with another pair of electrodes: Fp1–A1, Fp2–A2, Fpz–A1, and Fpz–A2 [51], where the Fpz acts as the ground (G), as illustrated in the first method. In the last 2 years, EEG signals were recorded from 17 different electrode locations according to the international 10–20 system, with a sampling rate of 200 Hz [34,92]. Single-channel equipment was recently developed and fabricated for collecting EEG data wherein only the Fz electrode is used as the common average reference in the montage.

### 5.2. Denoising EEG Signal Stage

**Figure 7.**
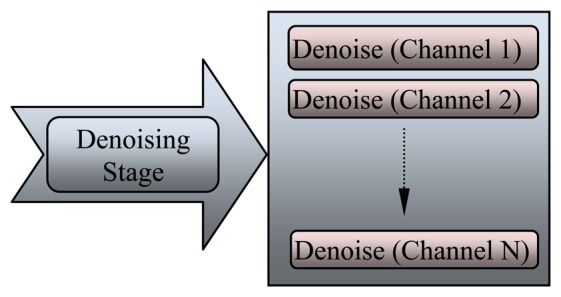
Multi-channel denoising stage to the recorded EEG signal.

The EEG signals are recorded with a lot of noise generated from the environment or artifacts. During the 1980s, digital filters were used in the initial stage of EEG data processing to remove power frequency (noise) from the observed signal and to reduce undesirable frequency components. The electrical line noise was removed directly from the EEG signal by a chain of low-pass and high-pass filters. Nitschke *et al.* [93] simulated and reported that the EEG signal diagnosed with the digital filter in time domain typically involves cross-multiplying each unfiltered data point and its neighbors with a set of weights. The second type of noise is the artifact, which appear as sharp waves, spikes, and spike-waves in the EEG signal because of movements of electrodes, head, and eyes (EMG signal). Artifacts may appear because of involuntary actions such as breathing, sweating, muscle activity, heartbeat, and eye blinks. Each channel should be processed and denoised separately from the others, as shown in Figure 7, which illustrates the denoising stage for each EEG channel.

**Figure 8.**
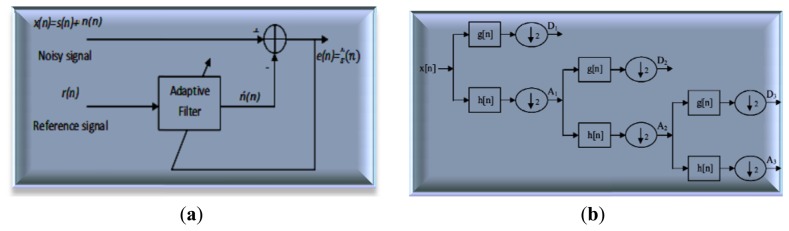
The principle of the Adaptive noise canceller and Wavelet levels. (**a**) Denoising using Adaptive noise canceller; (**b**) N-level of Wavelet denoising.

The discrete wavelet transform (DWT) removes various artifacts such as inherent noise, motion artifacts, and ocular artifacts, which are used to present the degree of variation in the EEG signals and reflect the effect of anesthetic drugs [94]. With the suitable choice of wavelet level and smoothing method, artifact noise can be removed to verify and analyze the EEG signal. Mother wavelet is particularly effective in describing various sides of non-stationary signals such as the discontinuities and repeated patterns of the recorded EEG signal. DWT is achieved by a successive chain of low-pass and high-pass filters in discrete time domain (adaptive filter). Figure 8(a) shows the principle adaptive filter used to extract noise from the EEG signal where this circuit is compatible with Equation (2).

The input signal *x*[*n*] is passed through a high-pass filter with impulse response *h*[*n*]. The same input is passed simultaneously through a low-pass filter with the impulse response *g*[*n*]. The detailed coefficients are given from the high-pass filter *y*_high_[*n*] and the approximation coefficients are given from the low-pass filter *y*_low_[*n*], as shown in Figure 8(b). The output filters (convolution) are given in Equations (4) and (5):

(4) $$y_{\text{low}}\left[n\right]=\sum_{k=-\infty }^{\infty }x\left[k\right]g\left[2n-k\right]$$

(5) $$y_{\text{high}}\left[n\right]=\sum_{\text{k}=-\infty }^{\infty }x\left[k\right]h\left[2n-k\right]$$

Wavelet transform (WT) has a basic formula, which can be used as the mother wavelet function. To use this transformation effectively, accurate details of the specific application should be considered and the suitable mother wavelet function should be chosen strictly. The final formula of the wavelet expression relative to the scaling function *φ*(*t*) and mother wavelet *ψ*(*t*) of a signal *x*(*t*) is as follows [95]:

(6) $$x(t)=\sum_{k}c_{j_{0}k}\varphi_{j_{0}k}\left(t\right)+\sum_{j=j_{0}}\sum_{k}d_{jk}\psi \left(t\right)$$

The first part in Equation (6) represents the approximation at the arbitrary starting scale *j*_0_, where the second part describes the summation of the details. Thus, the accurate mother wavelet function is chosen according to its compatibility with the EEG signal and the ability to process those signals in biomedical applications. The selection of accurate filters determines the possibility of reconstruction and the shape of the wavelet. The wavelet function is determined by the high-pass filter, which produces the detail coefficients of the wavelet decomposition. The scaling function is very similar to the wavelet function, but is determined by the low-pass filter, which is associated with the approximation coefficients of the wavelet decomposition. The results show that this approach is suitable for eliminating artifacts caused by eye movements and has the advantages of easy implementation, stability, and low computational cost [96].

**Figure 9.**
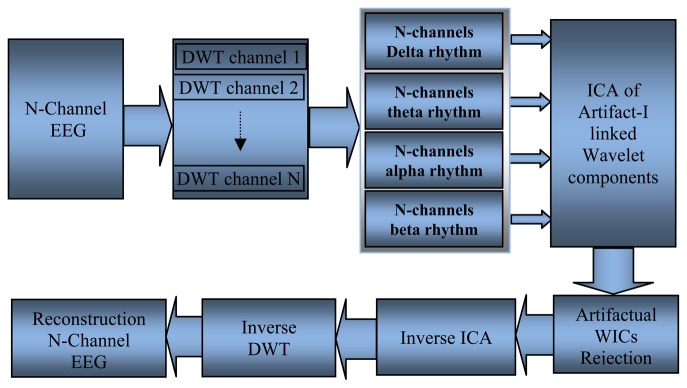
The combination structure between Wavelet transform and Independent Component Analysis.

Inuso *et al.* [97] and Walters-Williams and Li [98] used a new technique to remove the artifact from EEG signals; these researchers combined WT and independent component analysis (WICA), as shown in Figure 9. The proposed technique exhibited the best artifact separation performance for every kind of artifact and allowed minimal information loss. Another method used to remove ocular artifacts (EOG) and muscle artifacts (EMG) embedded with the recorded EEG signals is automatic artifact removal. SOBI was used to remove EOG artifacts, whereas canonical correlation analysis was used to remove muscle artifacts [99].

### 5.3. Feature Extraction of EEG Signal Stage

**Figure 10.**
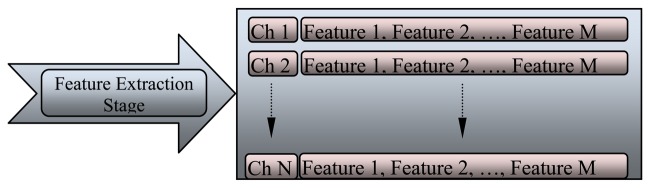
Multi-channel feature extraction stage.

To detect and monitor EEG variations during anesthesia, many features of EEG signal were identified to provide an automatic system that would support physicians during diagnosis. The classification stage cannot accept the recorded signal directly because of the huge amount of data that should be processed at one time, which slows down the classification system. The feature of each channel varies according to the location of the electrode on the scalp. The feature extraction stage is illustrated in Figure 10, where these features are arranged as an array. The array is subjected to many functions to generate a new array that represents the future of each channel.

Many researchers use the facility of the wavelet technique in analyzing the frequencies of brain signals and extracting multiple features. Zoughi and Boostani used WT to represent the basic content of EEG signals. The EEG signal is decomposed into different subbands according to the decomposition level, and then the energy of samples is calculated through each level. This method was proposed to extract the useful features from the recorded EEG signals during anesthesia. The WT decomposes an EEG signal into many frequencies in bands (mentioned in Section 3.2); thus, it is an effective tool for characterizing these signals. The EEG power and frequencies change constantly at each level of anesthesia within specific bands; the relationship between these variations in frequency band can be used to describe the DOA (mentioned in Section 4.4) [100]. Two types of features are used to detect the variation in EEG signal during anesthesia: the first one depends on variations in the power spectrum and the second depends on variations in the signal in the time-frequency domain. Recently, Li D. *et al.* used WT bicoherence to investigate the cross-frequencies coupling in the EEG signal with the concentration of the anesthetic agent isoflurane. Isoflurane caused two peaks; the first in the α range and the second in the δ range. Isoflurane caused cross-frequency coupling between α and slow δ waves. Increasing the concentration of isoflurane from 0.3% to 1.5% will shift the α peak frequency (11.3 Hz) to lower frequencies (7.1 Hz). In the same context, regarding the significant α peak that was phase-coupled to the slow δ waves, higher concentrations of isoflurane shifted this peak (10.8 Hz) to lower frequencies (7.7 Hz) [101].

The classic criteria for evaluating the various features are by calculating the mean squared error (Equation (7)) and the signal-to-noise ratio (Equation (9)). These values are calculated from the original EEG *x*(*n*) signal and the denoised EEG signal *x̂*(n) 97,102]:

(7) $$\text{MSE}=\frac{1}{N}\sum_{n=1}^{N}{\left[x(n)-\hat{x}(n)\right]}^{2}$$

(8) $$\text{SNR}=10\log\left|\frac{\sum_{n}{\hat{x}}^{2}(n)}{\sum_{n}{\left[x(n)-\hat{x}(n)\right]}^{2}}\right|$$

These values can be calculated at certain frequencies during anesthesia and compared with those during awareness to show the variations in values according to the DOA. The absolute power spectrum (power entropy) at specific frequencies (delta, theta, alpha, and beta) was used to find the maximum and minimum power values of the samples as well as the ratio of frequencies (delta/alpha and delta/beta) to monitor the variations in the EEG signals [103]. Srinivasan *et al.* used normalized spectral entropy to characterize the anesthesia levels. This spectral entropy was calculated for each EEG epoch within the efficient frequency range of brain signals. They used a short section (only 1 s) to monitor accurately the changes in EEG signal [104]. Approximate entropy (AE) and permutation entropy (PE) are proposed to measure the effect of anesthetic drugs using a stream of EEG data. These features reveal the effects of sevoflurane on brain activity. AE is based on the compatibility of events in phase space and is an appropriate method for defining the randomness of the system. AE depends on three parameters: *N*, the number of samples; *r*, the noise threshold; and *m*, the embedding dimension. PE is based on the Shannon entropy and is calculated using Equation (9) [105]:

(9) $$H_{p}\left(m\right)=-\sum_{j=1}^{J}P_{j}\ln p_{j}$$

where *P* is the probability distribution of the distinct symbols, which are defined as *p*_1_,*…*, *p_j_*; *m* is the permutation; and *J* should be less than *m*. Both PE and AE recognize the two statuses (awake and anesthetized) with high correlation to each other. The prediction probabilities show that PE has a stronger capability for differentiating between the two statuses. The results show that PE estimates the effects of sevoflurane more effectively than AE. This method can be applied to design a new EEG monitoring system for estimating the effects of sevoflurane [106].

### 5.4. Classification of EEG Signal Stage

**Figure 11.**
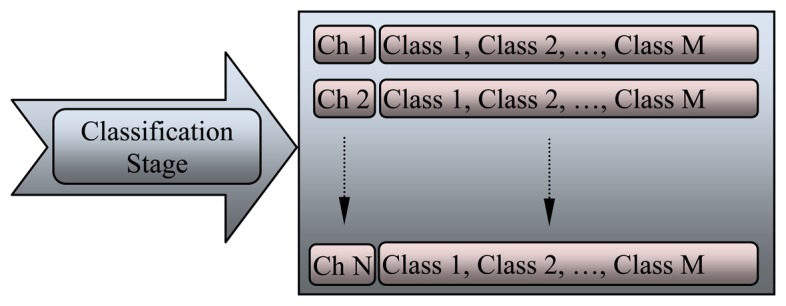
The classification stage for multiple channels.

The final stage in the processing and analysis of EEG signals is the classification stage. The features of EEG signals are extracted during the raw signal “feature extraction stage” and the redundant information has been reduced through “dimensionality reduction” in the previous stage. Distinguishing different categories among the process is necessary by applying a classifier. Figure 11 clarifies the classification stage for multiple channels, which can be used as a controller or indicator for DOA. Several techniques are used to classify EEG signals, such as neural networks (NN) classifier, linear discriminator analysis classifier, and support vector machine. These classifiers have different algorithms and accuracy rates. The algorithms depend on the methods used to teach the classifier, where accuracy depends on the clarity of the data, amount of the data, and the type of features that used in the classifier. Most of the classifiers need to be studied many times before they are used as such, however, the learning methods differ among classifiers. The data should be divided in three parts: the first dataset is for training the network and generating the hidden layer; the second dataset is for testing the performance of the classifier; and the third dataset is for finding and recognizing the results. The most popular method for detecting the DOA is the NN classifier because of its efficiency, accuracy, and applicability, with many groups of researchers recognizing the accuracy of the DOA system based on an artificial NN [107,108].

**Figure 12.**
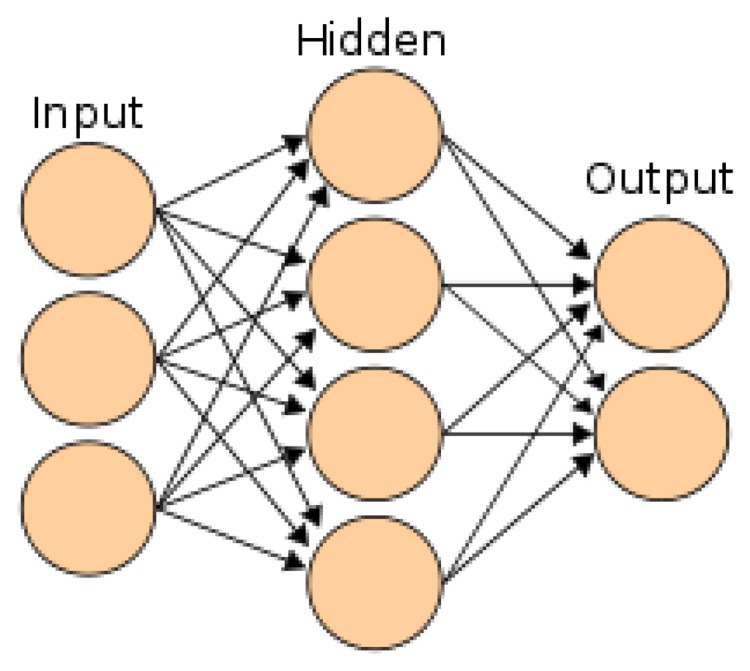
The interconnections of neural network with the group of nodes.

Artificial NNs are classifying systems that consist of a large number of simple high-interconnected processing elements called nodes or artificial neurons. This classifier is constructed similar to the structure and operation of the biological nervous system. The NN classifier learns through a special algorithm called “training.” Many types and architectures of NNs are fundamentally different from one another, depending on the network training method. Additional intermediate (hidden) processing layers should be used to solve the problems of nonlinearity and complexity. Figure 12 shows the typical structure and general stages of NN algorithms [109,110].

Many researchers introduced fundamental approaches for designing multilayer NN (MLNN) classifier models. The architecture of this classifier contains two or more layers. These two layers consist of an input layer containing the input variables, which represent the features extracted from EEG signals and the output layer containing the solution of the problem [111,112]. The cost function of MLNN is defined as follows:

(10) $$ɛ\left(n\right)=\frac{1}{2}\sum_{i=1}^{N}e_{i}{\left(n\right)}^{2}$$

(11) $$e_{i}(n)=d_{i}(n)-y_{i}(n)$$

where *d_i_*(*n*) and *y_i_*(*n*) are the desired and actual output of the *i*th output node of a network, respectively. Backpropagation algorithms are used to calculate the node weights. To normalize the input to NN, the mean value is subtracted from the input EEG signal and divided by the standard deviation (all inputs for training/testing set).

NNs can be used to analyze EEG signals to measure the DOA index, which is as informative as the BIS. Experiments confirm that when analyzing EEG data using NN achieves good discrimination between anesthetized and awake patients with good rejection of artificial signals is achieved. The flexibility and non-linearity of the NN approach are important factors for reliably monitoring the DOA [101]. Recurrent NN (RNN) is a powerful tool for classifying and modeling EEG. RNN consists of numerous simple computational units with weighted interconnections and delayed feedback connections. In this algorithm, all neurons in one layer are connected to all neurons in the next layer. These feedback connections provide RNN an intrinsic state and the ability to learn tasks that require memory [113,114]. The Elman RNN (ERNN) is also used to assess the DOA, which provides non-linear models for complex systems such as EEG signals, where the informative signals are too complex to be extracted by classic algorithms. This algorithm successfully estimates the amount of anesthetic gas that corresponds to the level of anesthesia [115]. Many researchers use the facility of the fuzzy technique in classifying the frequencies of brain during anesthesia. These researchers combined fuzzy logic and neural network to create an adaptive neuro-fuzzy diagnostic module. The proposed technique exhibited a substantial relationship between hypovolaemia and anesthesia during surgery [33,116].

## 6. Discussion

Physiologists have become accustomed to using the signal output of the brain as an index of brain health, as EEG signals provide a great deal of information about brain function. This review provides an overview of the EEG systems used to measure the DOA and the important stages that give clear signals that can be used by anesthesiologists to make correct decisions.

The design of EEG systems comprises four stages, namely, data acquisition, denoising, feature extraction, and classification. Different methods are used to clean EEG signals by removing artifacts. Some of these methods are suitable for removing artifacts such as HOS, ICA, WT, and linear filtering. A summary of the major methods is illustrated in Table 5. This table shows the advantages and disadvantages of each method in removing artifact noise. The researchers used two ways to monitor the DOA. The first method explains the brain waves recorded directly from the scalp. This method depends on the raw EEG signal by calculating EEG derivatives such as signal amplitude, power and frequency distribution, spectral entropy, and the correlations between recorded signals. These derivatives are used as features that are sent directly to the classification system.

The second method is EP, which stimulates the sensory organs of a patient and records the corresponding EEG signal. EPs include several ways of monitoring the DOA such as SEP, VEP, and AEP. EPs demonstrate the response of more localized areas of the midbrain, brainstem, and cerebral cortex to specific stimuli. EP represents the relationship between voltage and time, which can be quantified by measuring the inter-peak amplitudes and post-stimulus latency in the EEG signal. The comparison between three types of evoked responses is shown in detail in Table 6. AEP is widely used to estimate the DOA, which is divided into three main parts: the process of stimulus in the brainstem, the early cortical response, and the late cortical response. The first method is faster and easier than the second one because it does not need additional equipments and algorithms to monitor the DOA.

EEG signals are acquired from the frontal electrodes, those signals converted into several classifiable features. Thus far, no unified standard to the EEG features during anesthesia; these features depend on the variation of the amplitude, power, spectrum, bispectrum, entropy, approximate entropy and permutation entropy, *etc.* However, the features currently being used for monitoring the DOA achieve the desired purpose, but are insufficient for accurate, rapid, and definitive decision-making. Therefore, researchers are still currently attempting to find new EEG features that correspond to all cases, ages, and anesthetic agents.

Most researchers use NNs to classify the features of EEG signals because of their efficiency, accuracy, and applicability. Many types and architectures of NNs are fundamentally different from one another, depending on the network training method, number of hidden layers and type of outputs. Most monitoring devices currently use NN classifier, which refers to the number of training hours, number of layers, and estimated processing time.

Finally, many devices are used to describe the DOA during surgery. The BIS device represents the coupling of EEG frequencies and provides a range values from 0 to 100, which indicates deep anesthesia to consciousness. Another device is Narcotrend, which estimates and monitors the depth of unconsciousness using Kugler's classifier to classify EEG waves into different levels. The last one is Entropy, which is derived from non-linear dynamics and spectral entropy. Spectral entropy depends on the measure of information called Shannon entropy. This device calculates the response entropy within the frequency range (0 Hz to 47 Hz) with EEG and EMG activity. This device also calculates the state entropy for EEG activity within the frequency range from 0 Hz to 32 Hz. These algorithms are still unclear and have not been fully published. Numerous arguments support or reject these algorithms. Thus, no gold standard exists for estimating the level of unconsciousness after administering the anesthetic.

## 7. Conclusions

EEG signals carry valuable information regarding the brain system. This review aims to provide concise information about EEG signals during anesthesia and to reveal various methodologies for analyzing these signals. Techniques for EEG signal detection, decomposition, process, and classification were discussed, along with their advantages and disadvantages. Thus far, no monitoring systems are capable of measuring DOA and are compatible with all patients and all anesthetic agents, but the available monitors sufficiently provide good indication for doctors regarding the patient's condition in the surgical room. This study explains the various types of EEG signal analysis techniques during anesthesia. The right methods can be applied to the EEG signal to increase clarity, purity, and classification percentage for clinical diagnosis, biomedical research, hardware implementation, and end-user applications. The dynamic growth of microcomputer technology provides a greater scope for explaining observations of the anesthetic state in the future. If monitoring DOA becomes safe, simple, and economical, all anesthesia cases can be monitored easily.

## Figures and Tables

**Table 1.** Characteristics of intravenous anesthetic agents.

**Agent Name**	**Advantage**	**Disadvantages**	**Remark**
Propofol	(1) Excellent antiemetic properties. (2) It crosses the blood that going to the brain and redistributes quickly. (3) Excellent speed for inducing anesthesia and awakening the patient. (4) Great choice for short outpatient cases.	(1) Reduces the systemic vascular resistance and cardiac contractility that leads to a significant drop in blood pressure. (2) Depresses respiration in doses used for sedation and produces apnea in induction doses. (3) Causes a warm or burning sensation.	It's a lipid emulsion because it is not soluble in water, only in fat. The discomfort is decreased by giving first a dose of a local anesthetic.
Etomidate	Lack of a big blood pressure drops during surgery.	(1) Mostly postoperative nausea. (2) Can cause adrenal suppression with even a single dose (concern for patients who are on corticosteroids).	Very lipid soluble.
Thiopental	(1) Use for neurosurgery. (2) Crosses the blood-brain barrier quickly.	(1) Induces a decrease in cerebral blood flow due to vasodilation. (2) The patient may temporarily became much more dehydrated.	Very lipid soluble.
Ketamine	(1) Good bronchodilator, it's useful in asthmatics. (2) Doesn't cause apnea. (3) Good sedative for burn dressing change patients.	(1) Increases blood pressure and heart rate. (2) Causes dysphoric hallucinations. (3) Cause nausea.	It's an older anesthetic but still used. Generally patients are premedicated with midazolam.
Midazolam	(1) Potent sedative and anxiolytic (anxiety-relieving) and amnestic (memory-preventing) effects. (2) Use before going to the Operating Room.	Delayed wakeup compared with other induction agents.	Generally, used as benzodiazepines before the surgery.

**Table 2.** Characteristic of the Most Common Inhalational Agents.

**Name of agent**	**Advantage**	**Disadvantages**	**Remark**
Sevoflurane	(1) Fast. (2) Popular agent used with spine surgery.	(1) Smell not very good. (2) May cause kidney problems due to the high concentrations of Compound A (carbon dioxide absorbent). (3) Nothing's been proven till now.	To prevent overexposure to Compound A, anesthesiologists typically keep the fresh gas flow rate at 2 Lpm or higher
desflurane	(1) Fastest. (2) Great choice for long surgeries.	(1) May cause airway hyper-reactivity. (2) Cannot be used for mask induction to kids.	Not given to asthmatics or smokers.
Isoflurane	(1) Slow, great for patients who will remain intubated at the end of the intervention. (2) Used for heart surgeries and certain neurosurgical work. (3) Less expensive than other agents.	(1) Smell not very good. (2) Turns off early. (3) The monitoring catheter cannot be placed prior to and used during induction of anaesthesia.	Dilates the coronaries and cerebral vessels more than the other agents.
Nitrous oxide	(1) Very fast agent. (2) Odorless agent; its use for mask inductions in children or started with FiO_2_ before adding sevoflurane to avoid the bad smell.	(1) If the ratio is not balanced; FiO_2_ can enter to the lungs and cause hypoxia in the patient. (2) Poor choice for abdominal surgery and any area where air has been trapped (therefore dangerous in cases of pneumo-cephalus or pneumothorax).	Nitrous oxide: oxygen mixing ratio is 2:1 to avoid hypoxia; high-flow rate of oxygen given to the patient at the end of the intervention.

**Table 3.** The most common indexes used to monitor the depth of Anesthesia.

	**Index**	**Company**	**Index Range**	**Works with Agents**	**Not Work with Agents/Disadv.**
1	Bispectrum Index (BIS)	Aspect Medical Systems; Now Covidien, USA, 1992	0–100	Propofol, midazolam and isoflurane. Outperformed all.	Nitrous Oxide and ketamine. problems with EMG
2	Narcotrend Index NCT	MonitorTechnik, Germany, 2000	0–100	Children, sevoflurane propofol/remifentanil. EMG susceptibility Good artifact removal	Neuromuscular blocking agents Complex algorithm. Slowest response to a change in sedation.
3	Entropy Index	Datex-Ohmeda Company in 2003	0–100 1–91	Desflurane, sevoflurane propofol and thiopental	Ketamine
4	Patient State Index (PSI) or (PSA)	Physiomatrix, USA, 2001 Now SED Line Systems	0–100	Propofol, alfentanil, nitrous oxide EMG susceptibility	-
5	AEP-Monitor (AAI)	Danmeter, Denmark, 2001	0–100 OR 1–60	Propofol, midazolam and isoflurane	No effects of nitrous oxide and ketamine.
6	Snap Index	Everest Biomedical Instruments, USA, 2002	0–100	Sevoflurane and sevoflurane/nitrous Oxide	Sensitive to unintentional awareness
7	Cerebral State Index (CSI)	Danmeter A/S, Denmark, 2004	0–100	Propofol	Nitrous oxide

**Table 4.** The Algorithm Stages of Narcotrend Monitoring Device.

**Letter**	**State**
	
A	Awake
B	Sedated
C	Light anaesthesia
D	General anaesthesia
E	General anaesthesia with deep hypnosis
F	General anaesthesia with increasing burst suppression

**Table 5.** The advantages and disadvantages of artifact removal methods.

**Method**	**Advantage/Disadvantage**
Higher-order Statistics *HOS*	*HOS* methods may be used for analyzing the EMG signal due to its unique properties applied to random time series. The bispectrum or third order spectrum has the advantage of suppressing Gaussian noise. Carries the magnitude and phase information, which can be used to recover the system impulse function and input impulse sequence from the linear time-invariant *LTI* system output signal. *HOS* is blind to any kind of Gaussian process; a non-zero *HOS* provides a test of the extent of non-Gaussianity in a signal.
Independent Component Analysis *ICA*	Used when a large number of noises need to be distinguished. It's not suitable for on-line real time applications like *BCI*.
Wavelet Transforms *WT*	Linear method Represents a multi-resolution (frequency level) method. An alternative to other time frequency representations.
Linear filtering	Removes the artifacts located in certain frequency bands. *LPF* used to remove *EMG* artifacts and *HPF* used to remove *EOG* artifacts. Simple in design. The disadvantage is that it's not good when the frequencies of noises interfere or overlap with each other.

**Table 6.** Various Evoked potential methods that used to monitor DOA.

**Method**	**Method of Stimulation**	**Location of Stimuli**	**Location of Recorded Signal**	**Advantage**
Somatosensory evoked potential (SEP)	Electrical clicks	Somatosensory cortex	Somatosensory cortex	Monitor the response of tibial, peroneal or median nerve to stimulation
Visual evoked potentials (VEP)	Photic stimulation (using flashing lights)	Eyes	Occipital cortex.	Monitor function during surgery for lesions involving the optic nerve, pituitary gland and chiasma
Auditory evoked potential (AEP)	Audible clicks	Acoustic nerve	Primary auditory cortex	response to auditory canal stimulation
